# Supplementary material for: Polarization-transparent silicon photonic add-drop multiplexer with wideband hitless tuneability
Source: Nat Commun. 2021 Jul 15;12:4324. doi: 10.1038/s41467-021-24640-5 (PMC8282818; doi:10.1038/s41467-021-24640-5)
Supplement: Supplementary file 1 — Supplementary Information [file 41467_2021_24640_MOESM1_ESM.pdf]

Supplementary information

Polarization-transparent silicon photonic add-drop multiplexer with wideband hitless tuneability

Francesco Morichetti[1], Maziyar Milanizadeh[1], Matteo Petrini[1], Francesco Zanetto[1], Giorgio Ferrari[1], Douglas Oliveira de Aguiar*[1], Emanuele Guglielmi*[1], Marco Sampietro[1], and Andrea Melloni[1]

[1]Dipartimento di Elettronica, Informazione e Bioingegneria, Politecnico di Milano,

via Ponzio 34/5, 20133 Milano, Italy

Correspondence: F. Morichetti, E-mail: francesco.morichetti@polimi.it

(*) Now with PhotonPath s.r.l., via Durando 39, Milano, Italy

1. Numerical optimization of the FSR-free filter design

To increase the free spectral range (FSR) of a coupled MRR filter with rings of the same size, the bending radius of the MRRs must be reduced. To cover a wavelength range as large as 40 nm, bending radii below 3 μm would be required, which result in high radiation m and severe performance degradation. Moreover, the minimum size of the MRRs is also limited by the maximum temperature achievable by the thermo-optic actuators employed for the tuning. To realize filters operating on a broad wavelength range, Vernier schemes can be used [1, 2], where MRRs with different FSRs are employed. In these schemes, since the resonances of each MRR occur periodically forming a comb spectrum, they will overlap only at certain wavelengths, while suppressing all the resonances that lie in between. Typically, the ratios between the FSRs of the various MRRs are selected according to integer numbers and with this strategy tunable filters working across a wavelength range as large 32nm in [3] and 36.7nm in [4] were demonstrated.

In our filter we employed a modified Vernier scheme based on non-integer ratios between the FSRs of the MRRs [5], demonstrating that this modification can bring to a frequency response that is theoretically aperiodic (FSR-free filter). The schematic of the filter architecture is shown in Supplementary Fig. 1a. Two tunable couplers connecting the filter to the bus input/output waveguide allow the compensation of the wavelength dependence of the couplers along the band. The design procedure follows three main steps according to the flowchart of Supplementary Fig. 1b:

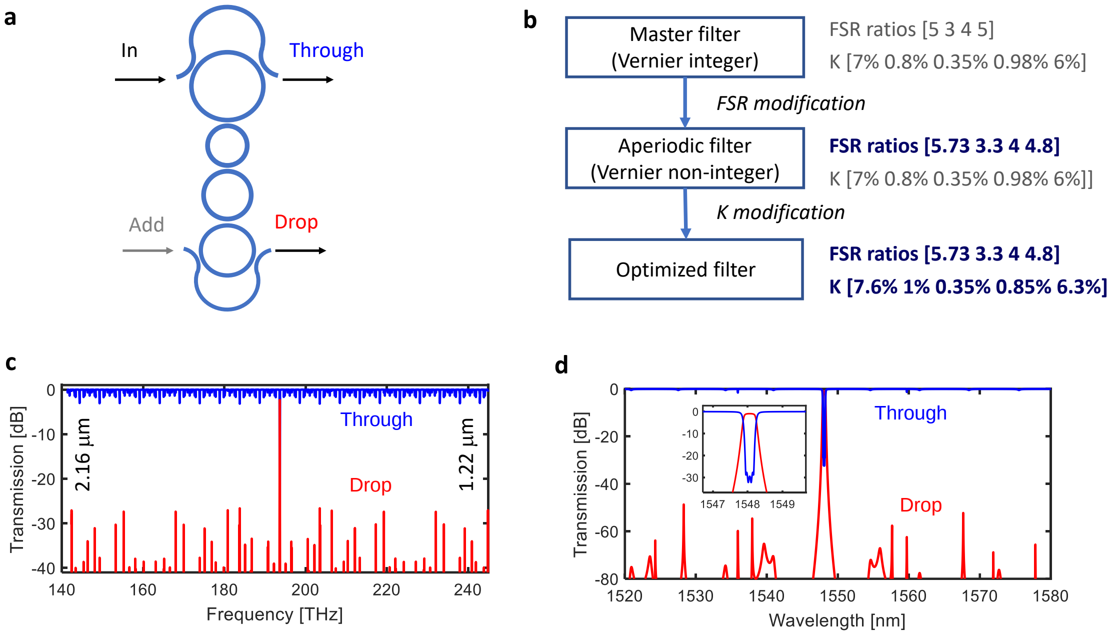

Supplementary Figure 1. (a) Schematic of 4th order Vernier filter with two tunable couplers connecting it to input/output bus waveguide. (b) Flowchart of design procedure, with FSRs and couplers values (the values of the power coupling ratios of the 1st MRR and the 4th MRR refer to the point couplers of the MZI). (c) Through and Drop port simulation of filter designed based on non-integer Vernier scheme neglecting the dependence of coupler on wavelength presenting more than 120THz of FSR. (d) Frequency response of 4th order filter with 40GHz bandwidth and more than 90nm of FSR.

1) **identification of a 4-th order master filter**. This is the seed of our optimization procedure and consists of a Vernier filter with integer FSR ratios. The design of this filter can be performed by using conventional techniques for the synthesis of coupled resonator filters [2]. Here, we considered as many as possible configurations for the FSRs of the four MRRs, which were supposed to be an integer fraction of the targeted FSR (4.8 THz) of the whole filter. This means that the FSR of the MRRs can assume values of 4.8THz/q_i with the integer q_i being conveniently comprised between 3 and 6. Among these combinations, we selected the configuration with integer ratios q_i = [5 3 4 5] (i = 1, 2, … 4) which guarantees the lowest off-band transmission peaks in the Drop port (> 30 dB) and the shallowest transmission notches in the Through port (< 1.5 dB). The coupling coefficients of the master filter K_i = [7%, 0.8%, 0.35%, 0.98%, 6%] provide a passband of 40 GHz, where the power coupling ratio of the 1st MRR (7%) and the 4th MRR (6%) refer to the value of each point couplers of the MZI.

2) **generation of a non-periodic filter**. The integer ratios q_i of the master filter are modified around their nominal value in order to improve the *off-band response* of the filter. A numerical optimization is performed targeting to cancel out the Drop port transmission at the passband replicas (4.8 THz from the nominal passband), while keeping low off-band transmission peaks in the Drop port and the shallowest transmission notches in the Through port. As a result,

a Vernier filter is achieved with non-integer ratios q_i = [5.73; 3.3; 4.0; 4.8] with a single transmission passband across a frequency range of more than 100 THz, as shown in Supplementary Fig. 1c. For this stage the coupling coefficients K_i are not modified with respect to the values found in the previous step (master filter) and are considered wavelength independent.

3) **filter optimization**. In the last step, the *in-band response* of the filter is optimized across the targeted wavelength range (1520 nm-1580 nm) by modifying the coupling coefficients K_i of the MRRs. To this aim the wavelength dispersion of the waveguide and of the directional couplers was taken into account (see Supplementary Sec. 2). For the optimization procedure, we considered the passband of the filter tuned at three different wavelengths (namely 1520 nm, 1545 nm and 1570 nm) and a cost function was defined to minimizing the spread of the spectral response with respect to the target filter specification (3 dB bandwidth of 40 GHz, 18 dB of Through-port in-band isolation, 20 dB Drop-port isolation at 50 GHz distance from the center of the filter). The simulated frequency response of the optimized filter with coupling coefficients K_i = [7.6%, 1%, 0.35%, 0.85%, 6.3%] (i = 1, 2, … 5) is shown in Supplementary Fig. 1d and key metrics are given in Supplementary Table 1. The off-band response of the optimized filter has Drop-port transmission peaks lower than -30 dB and Through-port notches of less than < 1.2 dB.

Supplementary Table 1: Performance of 4th order filter based on non-integer Vernier scheme.

Vernier Filter	**3dB Bandwidth**	**50GHz Channel Isolation**	**100GHz Channel Isolation**	**Max Drop Port Isolation**	**Max Out of Band Notch Depth**
Specification	40 [GHz]	20 [dB]	30 [dB]	18 [dB]	1.2 [dB]
@ 1520 nm	39.5 [GHz]	25.2 [dB]	53.2 [dB]	25 [dB]	1.2 [dB]
@ 1545 nm	43.9 [GHz]	22.5 [dB]	51 [dB]	28 [dB]	1.2 [dB]
@ 1570 nm	47.9 [GHz]	20.1 [dB]	50 [dB]	20.5 [dB]	1.2 [dB]

2. Directional coupler design

The cross section of the directional coupler of the MRRs is shown in Supplementary Fig. 2a. Supplementary Fig. 2b shows the top-view schematic of the coupler’s layout. The two coupled waveguides have a different curvature in the various directional couplers because of the Vernier scheme with MRRs with a different FSRs. According to the design parameters optimized in Supplementary Sec. 1, power couplings K_i = [7.6%, 1%, 0.35%, 0.85%, 6.3%] need to be realized among the MRRs with radii R_i = [14.6; 8.4; 10.2; 12.2] μm. Since symmetric (equal bending radii) and asymmetric (different bending radii) couplers are required, specific simulations were carried out by using a finite difference time domain (FDTD) tool to optimize the power coupling ratio and to investigate the wavelength sensitivity of the difference coupler configurations.

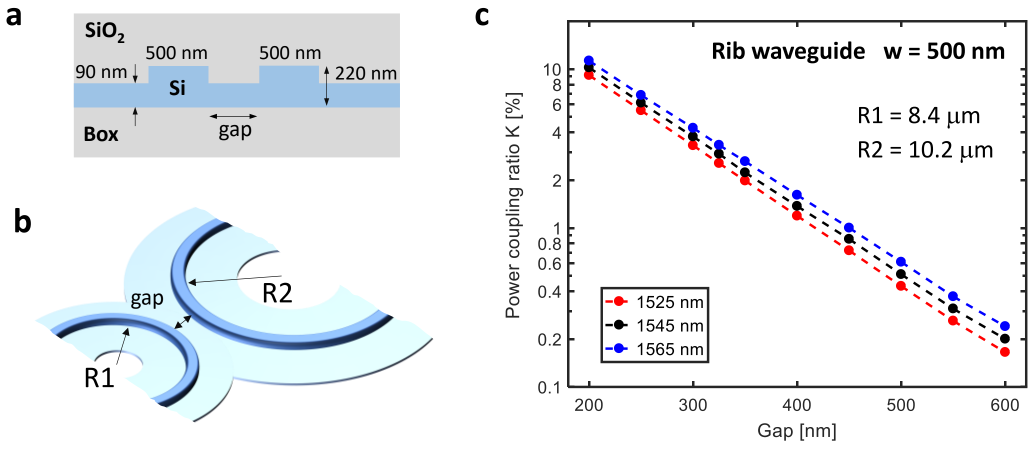

Supplementary Figure 2. Schematic of (a) the cross-section and (b) the top view structure of an asymmetric directional coupler with different bending radius $R_1 = 8.4$ µm and $R_2 = 10.2$ µm, implementing the inner directional coupler of the Vernier filter. (c) FDTD simulation (circles) of the power coupling ratio K of the asymmetric ring- ring coupler versus the gap distance at wavelengths (1525 nm, 1545 nm and 1565 nm). Dashed straight lines show the exponential fit of the simulated data.

To give an example, Supplementary Fig. 2c shows the power coupling ratio K of a directional coupler between bent waveguides with radii $R_1 = 8.4$ µm and $R_2 = 10.2$ µm versus the gap distance (between 200 nm and 600 nm) at three different wavelengths (1525 nm, 1545 nm and 1565 nm). A gap distance of about 530 nm is required to realize a coupling of 0.3% between the 2nd and 3rd MRRs of the filter.

The wavelength dependence of the inner directional coupler is shown in Supplementary Fig. 3. The wavelength sensitivity is defined as

$$S = \frac{K_{1565} - K_{1525}}{K_{1545}} \qquad \text{(Supplementary Equation 1)}$$

where K_{1565}, K_{1524} and K_{1525} are the power coupling coefficients at the maximum, mid and minimum wavelength across a 40 nm band. Results in Supplementary Fig. 3b show that the wavelength sensitivity of K increases with the gap distance. A minimum sensitivity $S = \pm 10.5\%$ is observed at the lowest gap (200 nm, corresponding to $K = 10\%$), while a maximum sensitivity of $S = \pm 18.5\%$ is observed at the largest gap 600 nm, corresponding to $K = 0.2\%$).

The coupling of the MRRs with the bus waveguides is controlled by using tunable Mach-Zehnder interferometers (MZIs) in order to optimize the performance of the filter over the whole wavelength range. To achieve the required power coupling ratio $K_{MZI1} = 28.1\%$ and $K_{MZI5} = 23.6\%$ with a tuneable MZI, the two point couplers must have a power coupling ratio $K_{1,5}$ of at least

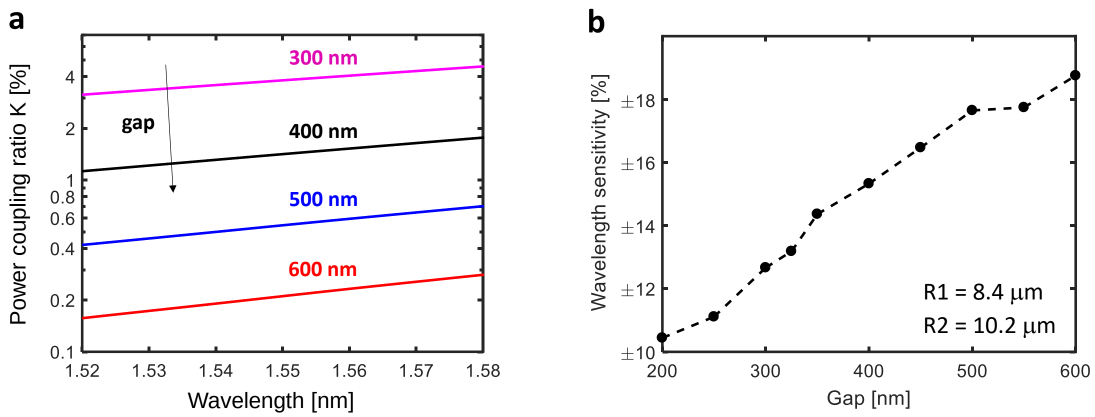

Supplementary Figure 3. FDTD simulation of the power coupling ratio K of the asymmetric ring-ring coupler (R_1 = 8.4 μm and R_2 = 10.2 μm) across the extended C band for increasing values of the gap distance. (b) Wavelength sensitivity of the power coupling coefficient versus the gap distance.

$$K_{1,5} = \sin^2\left(0.5 \sin^{-1}\left(\sqrt{K_{MZI1,5}}\right)\right) \qquad \text{(Supplementary Equation 2)}$$

that are $K_1 \geq 7.6\%$ and $K_5 \geq 6.3$ %. Supplementary Fig 4a shows the simulated power coupling ratio K for a directional coupler with radius R_1 = 13.7 μm and R_2 = 13.7 μm versus the gap distance (between 150 nm and 300 nm) at three different wavelengths across the extended C band (1525 nm, 1545 nm and 1565 nm). A gap distance of about 250 nm is required to have K_1 = 7.6% between the input bus waveguide and the first MRR of the filter. The wavelength sensitivity versus the gap distance is reported in Supplementary Fig. 4b.

The same numerical analysis was carried out for all the directional couplers of the filter.

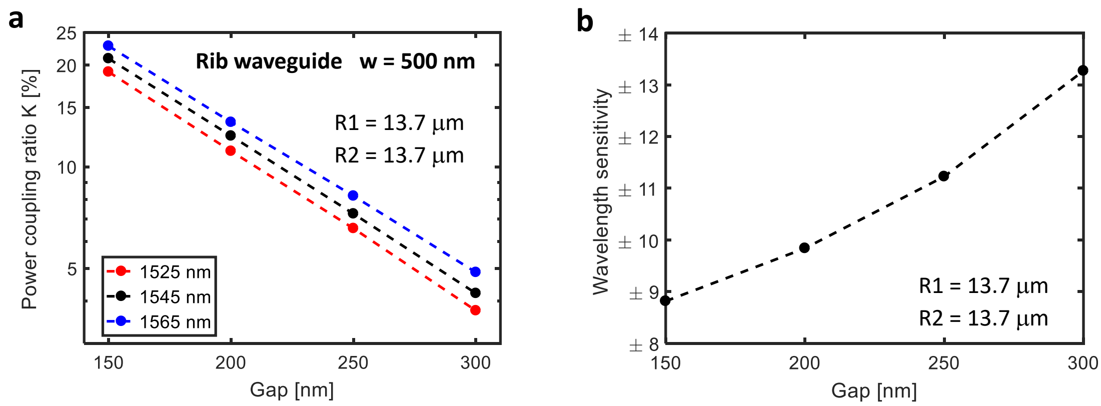

Supplementary Figure 4. (a) FDTD simulation (circles) of the power coupling ratio of the asymmetric directional coupler with radii R_1 = R_2 = 13.7 μm versus the gap distance at three different wavelengths (1525 nm, 1545 nm, 1565 nm) across the extended C band. Dashed straight lines show the exponential fit of the simulated data. (b) Wavelength sensitivity of the power coupling coefficient versus the gap distance.

3. Optical loss and electrical power consumption

Fiber-to-chip coupling is performed by using optimized mode adapters (suspended tapers [6] provided by silicon photonic foundry (AMF) and small core fibers (UHNA7, Nufern, https://www.nufern.com/pam/optical_fibers/988/UHNA7/#) with a mode field diameter (MFD) of 3.2 μm. Blue curves in Supplementary Fig. 5(a) show the insertion loss of silicon waveguides with a length of 0.5 cm and 7.1 cm across a wavelength range of 60 nm from 1520 nm to 1580 nm for TE polarized input light. From this measurement, we estimated a propagation loss of less than 1 dB/cm and a coupling loss of 3 dB/facet (red curve), with less than 0.2 dB wavelength dependent loss.

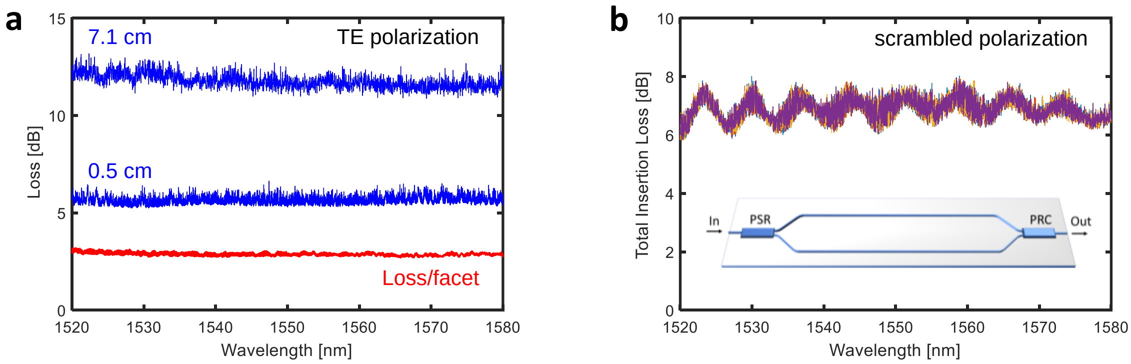

Supplementary Figure 5. (a) Insertion loss of 0.5 cm and 7.1 cm long waveguides (blue curves) and coupling loss of suspended tapers coupled with small core fibers (red curve) for TE polarization. (b) Total (fiber to fiber) insertion loss of the polarization diversity test structure, when the polarization state of the input light is randomly scrambled during the wavelength scan.

The total loss of the polarization diversity scheme was assessed by measuring the insertion loss of the test vehicle shown in the inset of Supplementary Fig. 5(b), including the I/O mode adapters (suspended tapers), a PSR/PRC pair and 5-mm-long bus waveguide. The average insertion loss measured across a 60 nm wavelength range around the extended C band is about 7 dB. The four overlapped curves, which are measured by scrambling the polarization during the wavelength scan, show a PDL of less than +/- 0.6 dB. The on-chip loss of the polarization diversity scheme (PSR, PRC, propagation in the bus waveguides) is less than 1 dB in total.

Regarding the electrical power consumption, the tuneable filter device is controlled by 8 independent controls, providing the thermal tuning of the 4 MRRs of the filter, the thermal tuning of the 2 MZIs tuneable couplers and the loss control of the 2 VOAs integrated in the inner MRRs of the filter.

The heater integrated in a MRR with no VOA requires about 31 mW to provide a π-shift, while in a MRR with integrated VOA the electrical power required for introducing a π-shift raises to about 46 mW, because the doped-silicon slab surrounding the MRR and the metallic lines required to feed current into the VOA are responsible for heat dissipation outside the core of the waveguide. The MZI tuneable couplers of the filter need to be thermally controlled within a π phase shift, this requiring about 30 mW each.

Regarding the VOA, the complete disconnection of the filter requires a round trip loss of 4 dB in both inner MRRs. This effect is achieved when the VOA is fed with a driving current of 25 mA (3 V), resulting in a dissipated electrical power of 75 mW for each VOA.

As a result, the complete reconfiguration process of the filter requires in the worst case (2π phase shift to all the MRRs and π-shift to both MZIs) a peak power dissipation of about 370 mW for the thermal tuning (124 mW for the tuning of the 1st and 4th MRR, 184 mW for the tuning of the inner MRRs, 60 mW for the control of the MZI tunable couplers) and an energy consumption of less than 15μJ for the filter disconnection through the switching on of the two VOAs (150 mW power for less than 100μs).

4. VOAs integrated in silicon MRR

The impact of the VOA integrated into a MRR was experimentally investigated by measuring the frequency response of single MRR with different VOA designs. As shown in Supplementary Fig. 6a we define as clearance the distance *d* of the p^{++} and n^{++} doped regions in the lateral slab from the 220-nm-thick region of the silicon waveguide core. The same figure shows the 3D schematic of the MRR with doped regions, electrodes and VIAs. Supplementary Figure 6b shows the experimental round trip loss versus the clearance distance as extracted from the numerical fit of the MRR frequency responses. For example, Supplementary Fig. 6c shows the Drop port (blue curve) and Through port (red curve) transmission of MRRs with *d*=0.3 μm (a), 0.9 μm (b), and 1.1 μm (c). The insertion loss at the Through port response is due to fiber-to-chip vertical coupling with conventional grating couplers, introducing a loss of about 3-4 dB/grating. Results show that for a clearance of 1.1 μm, the round-trip loss is as low as 0.015 dB/turn, this number being in line with the round-trip loss of a MRR with no integrated VOA. Reducing the clearance to 0.9 μm, a slight increase of the MRR loss is observed, this value being still below 0.02 dB/turn. For clearance values below 0.5 μm the round trip loss increases by one order of magnitude (0.19 dB/turn), thus affecting significantly the MRR response because of the overlap of the guided mode with the highly doped regions of the VOA (n^{++} and p^{++} in the lateral slab).

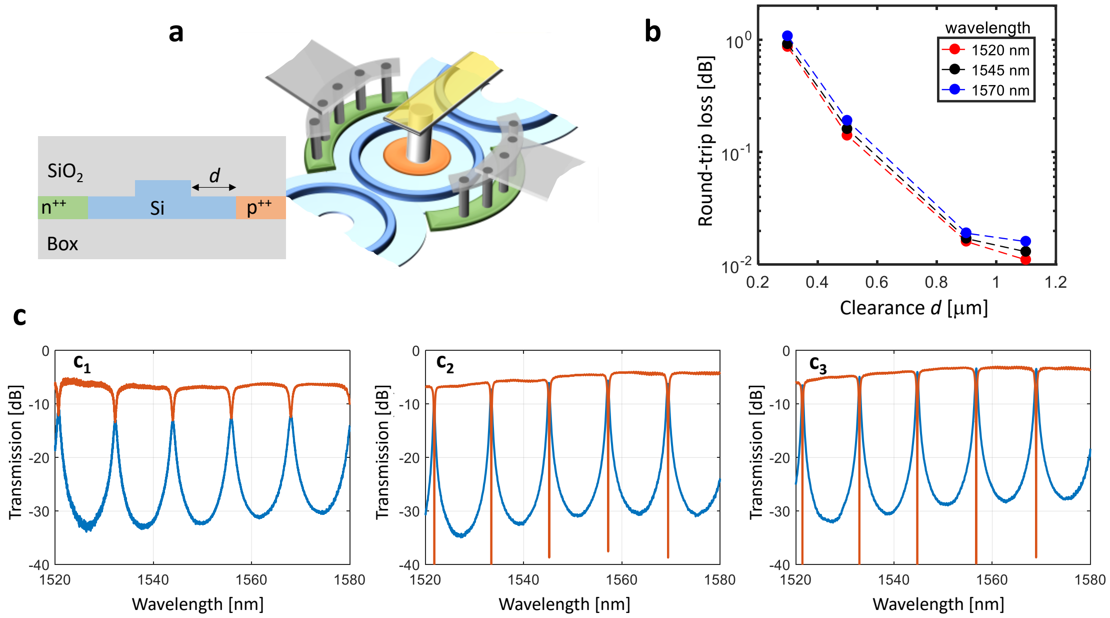

Supplementary Figure 6. (a) 3D schematic of a ring resonator with VOA doped regions, electrodes, VIAs and waveguide cross section. The clearance is indicated with *d*. (b) Experimental round-trip loss for three different wavelengths. (c_1-c_3) Measured Through port (red curve) and Drop port (blue curve) transmission of single MRR filters versus the clearance distance of the VOA: 0.3 μm (a), 0.9 μm (b), and 1.1 μm (c).

The hitless strategy employed for the tuning of the proposed filter exploits the control of the MRR roundtrip loss through VOA, which are integrated in the 2nd and 3rd MRR of the filter. To assess the performance of VOAs integrated in the MRR, we measured the spectrum of a single MRR filter when the forward voltage applied to the VOA is increased. Supplementary Figure 7 shows the Through port transmission (a) and the Drop port transmission (b) when the VOA voltage increases from 0V to 2.5V. Because of the free carriers injected into the core of the waveguide, the round-trip loss progressively increases, resulting in a reduction of the notch at the Through port and a reduction of the transmission peak at the Drop port. When the voltage exceeds 2.5 V (green curve), the Through port notch is around 1 dB and more than 14 dB isolation is achieved at the Drop port. From a numerical fit of the MRR transmission at different voltages, we derived the loss vs voltage curve shown in Supplementary Fig. 7c. The round-trip loss of the MRR increases to about 1 dB for an applied voltage of 2 V and up to 4 dB/turn for a voltage of 3V. According to the numerical simulations reported in Supplementary Sec. 4, such a loss change is high enough to guarantee hitless tuning of the filter.

The loss increase is associated with a blue shift of the MRR transfer function. Since a blue shift is due to free carrier dispersion (FCD), while a red shift is induced by waveguide heating, we conclude that in our device FCD is the dominant effect in the entire range of operational voltages (0 - 3 V). The dynamics of loss modulation and FCD in the VOA occur in a time scale of about 1 ns, that is fast enough to be compliant with the switching time required in most optical networks. In contrast, thermal effects are orders of magnitude slower (tens of microsecond) and must be compensated, as discussed in Supplementary Sec. 5.

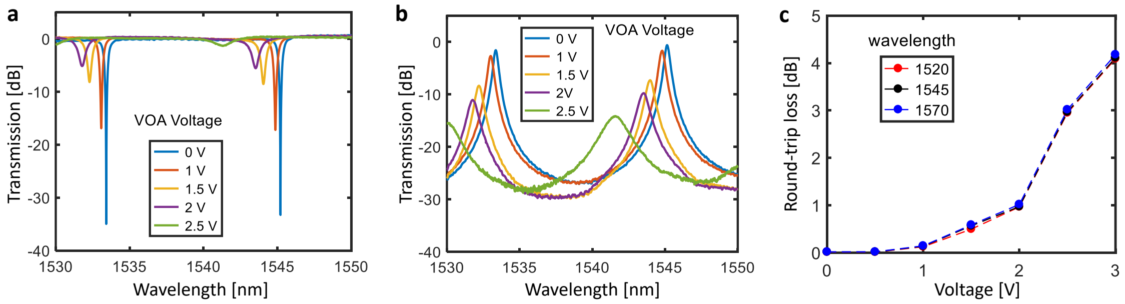

Supplementary Figure 7. Measured transmission at (a) Through port and (b) Drop port of a MRR integrating a VOA at increasing driving voltage. (c) Round trip loss of the MRR at increasing values of the VOA voltage.

5. Numerical simulation of hitless tuning

The hitless tuning of the proposed filter is performed by controlling the round-trip loss α of the MRRs [5]. The key point to implement a truly hitless scheme is that loss should not be introduced in the first MRR of the filter, as illustrated in Supplementary Fig. 8. Simulations in Supplementary Fig. 8a show the change of the Through port (blue lines) and Drop port (red lines) transmission of the filter of Supplementary Fig. 1d when the round-trip loss α of the first MMR is increased. When α = 1.5 dB, the MRR approaches the critical coupling condition and deep notches (30 dB) appear in the Through port transmission at a distance from the filter passband equal to the FSR of the first MRR. Note that this condition occurs well before the disconnection of the filter passband, which is attenuated by only 5 dB (lightest red line, 1548nm). By further increasing α, the notches reduce and the filter is correctly disconnected but the transition cannot be considered hitless.

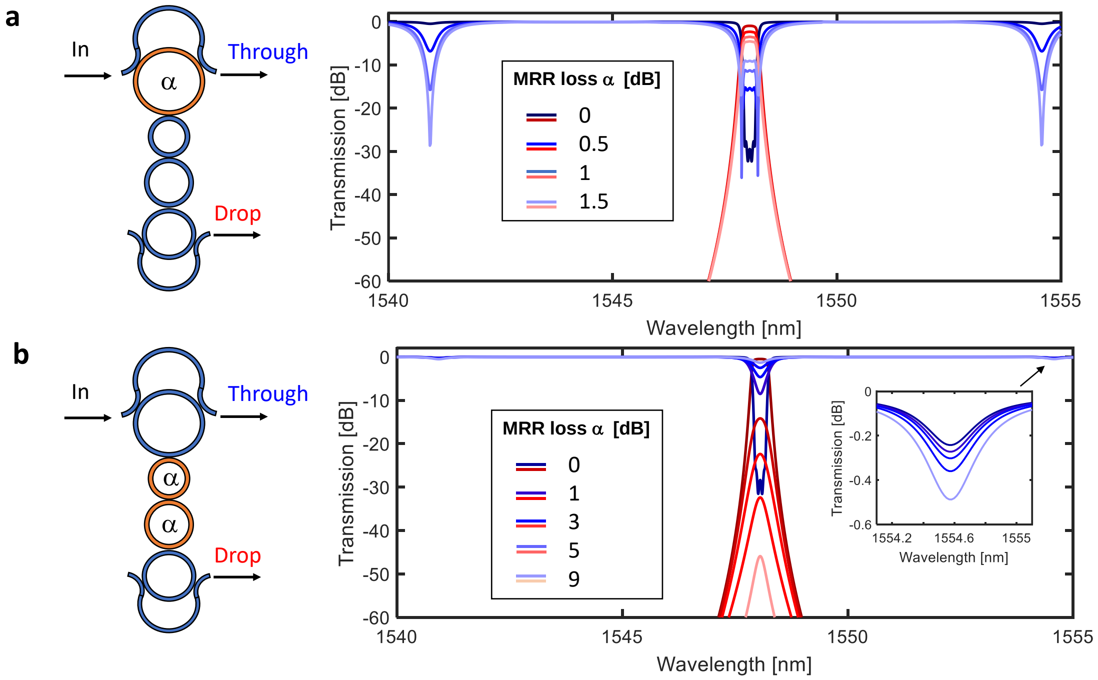

Supplementary Figure 8. Numerical simulations showing the change of the Through port (blue lines) and Drop port (red lines) spectral response of the filter when additional losses are introduced in (a) the first MRR of the filter and (b) the 2nd and 3rd MRRs of the filter. Detail of the notch at the Through in the inset.

A truly hitless disconnection is achieved by attenuating the inner rings of the filter. In principle only the second MRR of the filter could be switched off by increasing its roundtrip loss, but this would require a high attenuation per unit length that is difficult to achieve in the small footprint of a silicon MRR. Therefore, for the filter disconnection we consider the integration of p-i-n VOAs both in the 2nd and 3rd MRRs. Supplementary Figure 8b shows the simulated frequency response of the filter at increasing round trip loss. Complete disconnection of the filter from the

bus waveguide (> 35 dB Drop port isolation) is achieved when the additional round trip loss in both MRRs is about 5 dB. The in-band notches at the Through port remain less than 0.6 dB deep during the entire switching process as shown in the inset.

6. Thermal compensation of VOA-induced waveguide heating

Loss modulation in the silicon MRR can be controlled in a time scale of a few nanoseconds thanks to the fast response of the p-i-n VOA. The black curve in Fig. 2e of the main text shows the voltage signal of the photodetector (PD) at the output of a silicon MRR (same device as in Supplementary Figs. 5 and 6) when the voltage (red curve) driving the integrated VOA is increased. A fast drop of the light power is observed when the VOA is switched on (that means loss increase), which is mainly limited by the rise time of the voltage signal (about 40 ns). When the voltage driving the VOA is switched off, loss reduction is almost as fast as in the switch-on process, but the resonance of the MRR is detuned because of the waveguide heating caused by the dissipation of electrical power during VOA operation. As shown in Supplementary Fig. 9, the thermal time constant of the cooling down of the optical waveguide is in the order of 100 μs. This effect is the strongest limiting factor to the speed of the hitless tuning mechanism.

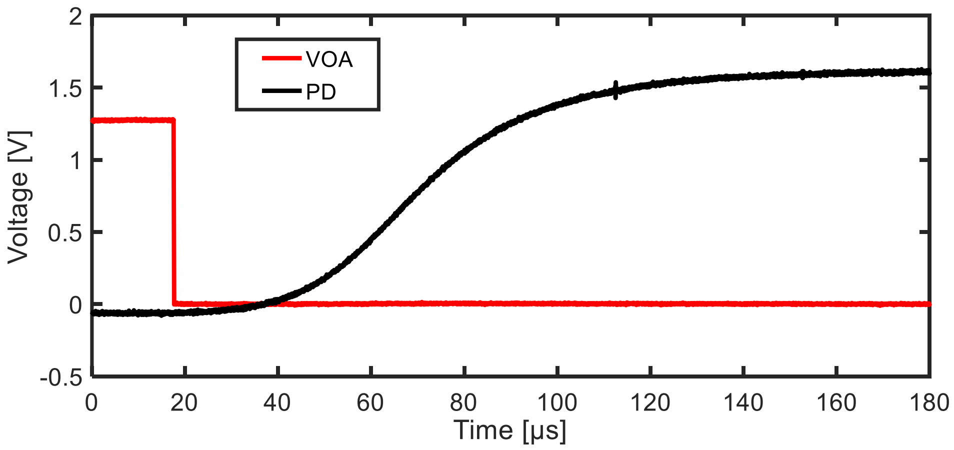

Supplementary Figure 9. Time response of VOA turning-off process with no thermal compensation performed by the integrated heater (PD = photodetector connected to the MRR output port).

To overcome this problem, we exploited the thermal tuners integrated in the MRRs of the filter to compensate for the slow-down induced by the VOA waveguide heating. To this aim we followed this procedure for the tuning:

1. *Filter disconnection*: switch-on the VOA by applying a fast step voltage to the VOA only.

2. *Heater activation*: add a “cooling down” bias voltage to the thermo-optic actuator to compensate for the heat generated by the VOA. This additional voltage does not introduce any optical perturbations to the filter response because the MRR is highly lossy and the filter is disconnected from the input bus waveguide.
3. *Filter connection*: switch-off the VOA by applying a fast step voltage to the VOA and simultaneously remove the “cooling down” bias to the thermo-optic actuator.

As shown in Fig. 2f of the main text, active compensation of the VOA-induced waveguide heating enables to reduce the time response of the switch-off process by more than two orders of magnitude (from 100 μs to about 400 ns). We expect that the time response can be further improved by optimizing the temporal shape of the cooling down voltage signal driving the thermal tuner [7] and its synchronization with the VOA operation.

7. Experimental setup for polarization diversity filter

In this section, we illustrate the experimental setup employed for the polarization sensitive measurements reported in the main text.

As shown in the block diagram of Supplementary Fig. 10a, the wavelength response of the filter is measured by using a tunable laser source (TLS, ANDO AQ4320A), operating in the 1520 nm - 1620 nm wavelength range, which is synchronized with an optical spectrum analyzer (OSA, ANDO AQ6317). The light is coupled into/out of the device through small core fibers (3.5 μm, mode field diameter) which are coupled to the silicon waveguide through suspended tapers [8]. The filters are actively controlled by a dedicated control board implementing the calibration and control algorithms described in [9].

A polarization scrambler (Thorlabs DPC 5500, mounted on a chassis TXP 5016) is inserted before the silicon PIC generating at its output random uncorrelated polarization states every 890 μs. Since each trace of the filter spectrum is acquired by sweeping the TLS at a speed of 6 pm/s, the polarization state in the data point of Fig. 3(c) of the main text and Supplementary Fig. 10 (b)-(c) (accounting for 16667 wavelength data points, 6 pm sampling step), are almost uncorrelated.

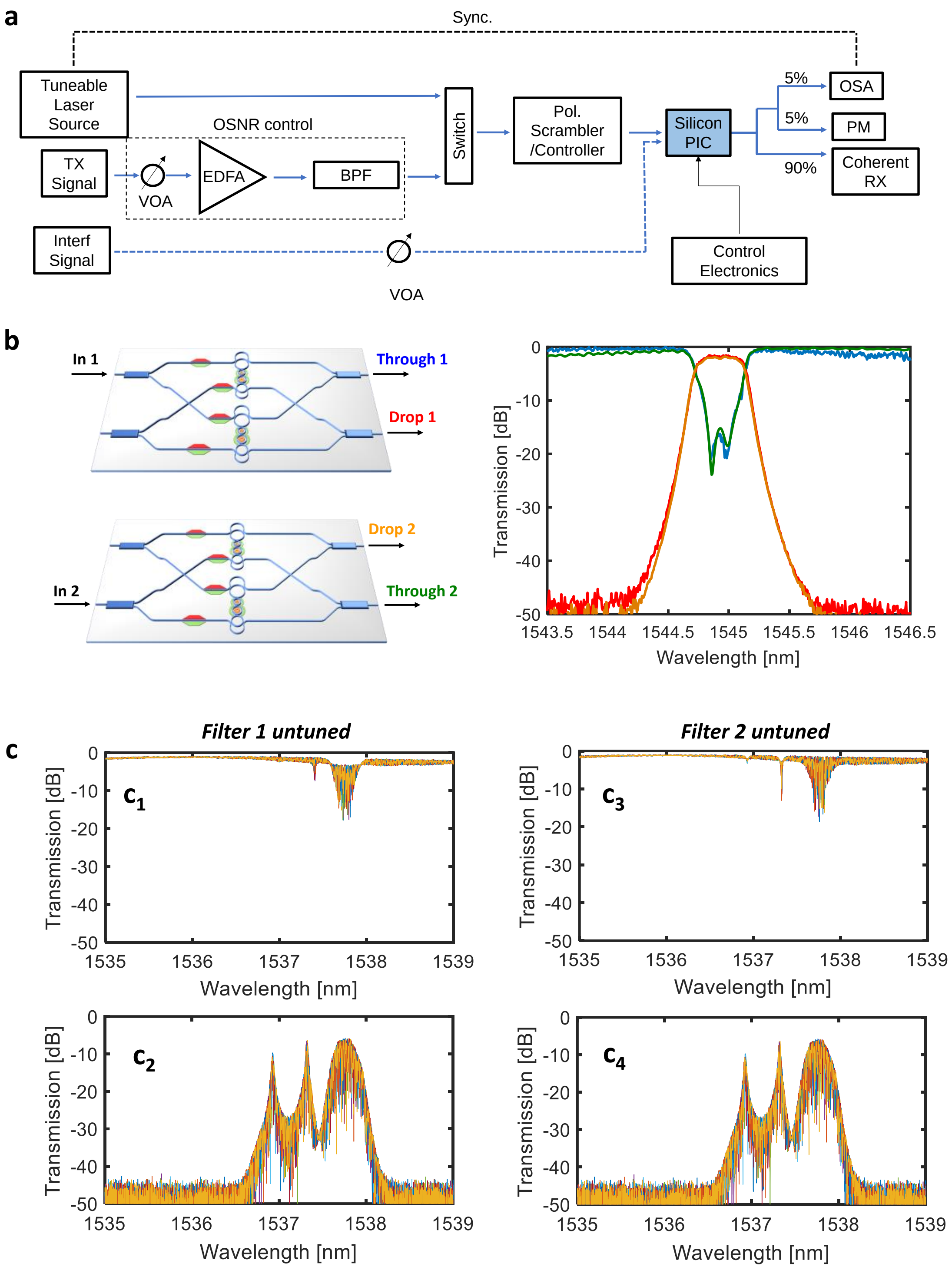

Supplementary Figure 10. (a) Experimental setup employed for calibration and testing of the polarization-diversity filter. (b) Spectral response of the filter (tuned around a wavelength of 1545 nm) showing the same Through port and Drop port behavior when the input signal is provided at different input ports (In1 and In2) of the filter. (c) Through port (c_1, c_3) and Drop port (c_2, c_4) spectral response of the polarization diversity filter when Filter 1 is untuned (left panels) and when Filter 2 is untuned (left panels). The two narrow peaks in the Drop port responses around 1537 nm belongs to the untuned filter, while the broader envelope of the spectral response around 1537.8 nm is the transmission of the tuned filter. Each point of the curves is associated with a different (random) polarization state.

Bit error rate (BER) measurements were performed by using the same experimental setup. We used a commercial transceiver (Jabil Photonics CFP2-DCO) generating 100 Gbit/s double-polarization QPSK signal and 200 Gbit/s double-polarization 16-QAM signal. The FEC threshold of the transceiver is 2e-2. In both cases the output power is about -2 dBm, the bandwidth is 32 GHz, and the central wavelength can be tuned along the C-band according to the 50-GHz spacing ITU-T grid. The transmitter is coupled to a variable optical attenuator (VOA), an erbium-doped fiber amplifier (EDFA, Optocom/Keopsys C-27-PB), working in the saturation condition with 16 dBm output power, and a 2-nm-wide band-pass filter. These devices were used to control the optical signal-to-noise-ratio (OSNR) of the transmitted signal. An optical switch is introduced to pass from the frequency domain measurement (TLS and OSA) to BER measurement. The output of the optical switch is directly coupled to a polarization scrambler/controller feeding the light to the silicon photonic integrated circuit (PIC) hosting the proposed filter. The output signal is split in different branches in order to allow power monitoring (5%), spectral measurements (5%) and BER measurements (90%). In all the presented results, the received power is -9 dBm.

The transceiver hosts a coherent receiver assisted by a digital signal processor (DSP) that can compensate chromatic dispersion (CD) up to 40000 ps/nm (100G signal) or 10000 ps/nm (200G signal), polarization dependent loss (PDL) up to 3 dB, polarization mode dispersion (PMD) of 15 ps. Furthermore, it can track changes of the state of polarization (SOP) of the light of 300 krad/s.

The interfering signal used in the experiments is a 100 Gbit/s double-polarization QPSK signal (28 GHz bandwidth, 5 dBm output power) generated by a different transceiver. The power of the interfering signal is controlled through a VOA in order to equalize it to the signal under test. In the reported experiment the BER was measured at the Drop port of the filter when the test signal is coupled to the In port and the interfering signal to the Add port, whereas the BER was measured at the Through port when the test signal is coupled to the Add port and the interfering signal to the In port.

8. Impact of out-of-band chromatic dispersion

For the channels transmitted at wavelengths outside the passband of the filter, the first MRR acts as an all-pass filter introducing some degree of chromatic dispersion (CD), whose impact needs to be carefully investigated.

Numerical simulations reported in Supplementary Fig. 11(a) show that the CD introduced by the out-of-band all-pass filter is less than 40 ps/nm, that is less than the CD caused by 3 km of standard fiber (17 ns/nm·km for G.652 fiber). As written in the Methods ("Filter quality assessment") the commercial transceiver that we used (Jabil Photonics CFP2-DCO) can compensate a CD of up to 40000 ps/nm (100 G signal) or 10000 ps/nm (200 G signal), that is more than two orders of magnitude higher. To prove the negligible impact of the out-of-band CD, BER measurements were performed on all the ITU-T WDM channels transmitted outside the passband of the filter within the extended C-band. Supplementary Figure 11(b) shows the BER curves for some of the measured channels, namely the ITU-T channel 47 (1539.77 nm), 40 (1545.32 nm), 34 (1550.12 nm), 29 (1554.13 nm), and 22 (1559.79 nm). No OSNR penalties due to dispersion effects were observed.

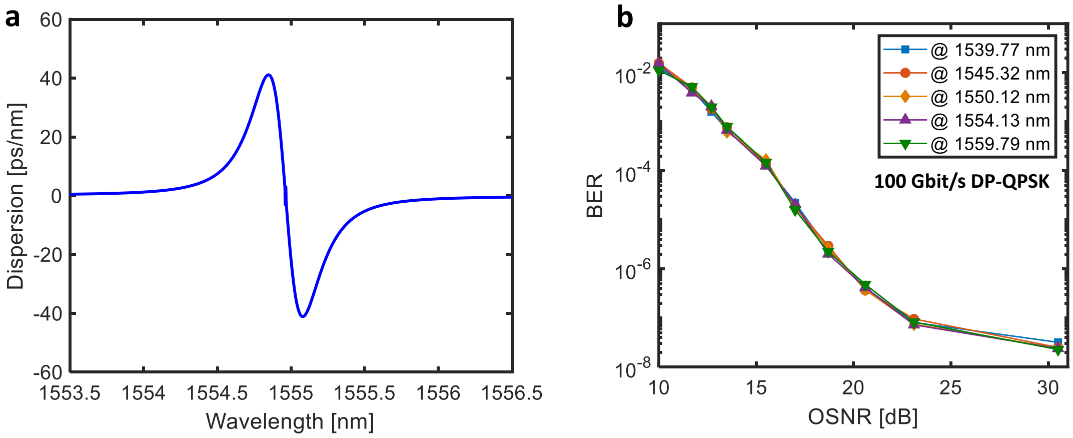

Supplementary Figure 11. (a) Numerical simulations of the chromatic dispersion introduced by first ring of the filter acting as an out-of-band all-pass filter; (b) BER curve of 100 Gbit/s DP-QPSK signals transmitted at several carrier wavelengths along the off-band wavelength range of the filter.

During the tuning process, dynamic changes in chromatic dispersion may also arise due to the overlap of the resonance of the first MRR (that is tuned without being disconnected) with adjacent channels. In principle, this time-varying oscillation in chromatic dispersion could be below the timescale for a transceiver to correct and it needed to be specifically investigated. Referring to the case of Fig 3d, we transmitted a 100 Gbit/s DP-QPSK signal with carrier

wavelength 1540.56 nm (ITU-T channel 46) and we measured the BER versus time during the switching of the filter from channel 60 (1529.55nm) to channel 34 (1550.12nm). As shown in Supplementary Fig. 12, once the filter is disconnected from the bus waveguide, the wavelength switch is operated after 5 s on a time scale of less than 10 µs, as given by the time response of the thermal tuners. The transceiver provides an updated BER measurement every 1 s over a time widow of 10 s. Results show no changes in the measured BER (pre-FEC) that remains below 2e-8 (OSNR 30 dB) during the entire tuning process. This means that the dynamic changes in the chromatic dispersion (as well as in the loss) are within the dynamic compensation margin of the transceiver and are negligible for the system performance.

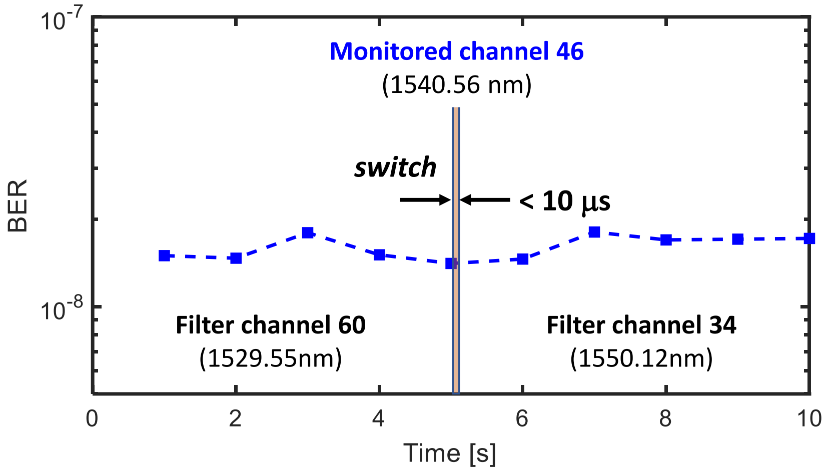

Supplementary Figure 12. Pre-FEC BER of a 100 Gbit/s DP QPSK channel transmitted at a carrier wavelength of 1540 nm (ITU-T channel 46) during the hitless tuning of the filter from channel 60 (1529.55nm) to channel 34 (1550.12nm).

References

[1] G. Griffel, "Vernier Effect in Asymmetrical Ring Resonator," IEEE Phot. Technol Lett., vol. 12, no. 12, 2000.

[2] A. Melloni and M. Martinelli, "Synthesis of Direct-Coupled-Resonators Bandpass Filters for WDM Systems," Journal of Lightwave Technology, vol. 2, pp. 90-103, 2002.

[3] Y. Ren, D. Perron, F. Aurangozeb , Z. Jiang, M. Hossain and V. Van, "Silicon Photonic Vernier Cascaded Microring," IEEE Phot. Technol Lett., vol. 31, no. 18, 2019.

[4] H. Jayatilleka, R. Boeck, M. AlTaha, J. Flueckiger, N. A. F. Jaeger, S. Shekhar and L. Chrostowski, "Automatic Tuning and Temperature Stabilization of High-Order Silicon Vernier Microring Filters," in Proc. Optical Fiber Communications Conference and Exhibition (OFC), 2017.

[5] M. Milanizadeh, M. Petrini, F. Morichetti and A. Melloni, "FSR-free filter with hitless tunability across C+L telecom band," in OSA Advanced Photonics Congress (AP) 2020 (IPR, NP, NOMA, Networks, PVLED, PSC, SPPCom, SOF), 2020.

[6] Q. Fang, J. Song, X. Luo, X. Tu, L. Jia, M. Yu and G. Lo, "Low loss fiber-to-waveguide converter with a 3-D functional taper for silicon photonics," IEEE Phot. Technol Lett., vol. 28, no. 22, pp. 2533-2536, 2016.

[7] M. Harjanne, M. Kapulainen, T. Aalto and P. Heimala, "Sub-μs switching time in silicon-on-insulator Mach-Zehnder thermooptic switch," in IEEE Photonics Technology Letters, vol. 16, no. 9, pp. 2039-2041, Sept. 2004.

[8] Available in the PDK of AMF, Advanced Micro Foundry, http://www.advmf.com, Singapore.

[9] M. Milanizadeh, S. Ahmadi, M. Petrini, D. Aguiar, R. Mazzanti, F. Zanetto, E. Guglielmi, M. Sampietro, F. Morichetti and A. Melloni, "Control and Calibration Recipes for Photonic Integrated Circuits," Journal of Selected Topics in Quantum Electronics, vol. 26, no. 5, pp. 1-10, 2020.
